# Prey size diversity hinders biomass trophic transfer and predator size diversity promotes it in planktonic communities

**DOI:** 10.1098/rspb.2015.2129

**Published:** 2016-02-10

**Authors:** Carmen García-Comas, Akash R. Sastri, Lin Ye, Chun-Yi Chang, Fan-Sian Lin, Min-Sian Su, Gwo-Ching Gong, Chih-hao Hsieh

**Affiliations:** 1 Institute of Oceanography, National Taiwan University, No. 1, Sector 4, Roosevelt Road, Taipei 10617, Taiwan; 2 Institute of Ecology and Evolutionary Biology, National Taiwan University, No. 1, Sector 4, Roosevelt Road, Taipei 10617, Taiwan; 3 Marine Ecosystem Dynamics Research Group, Research and Development Center for Global Change, Japan Agency for Marine-Science and Technology (JAMSTEC), 3173-25, Showa-machi, Kanazawa-ku, Yokohama, Kanagawa 236-0001, Japan; 4 Ocean Networks Canada, University of Victoria, Victoria, British Columbia, Canada; 5 State Key Laboratory of Freshwater Ecology and Biotechnology, Institute of Hydrobiology, the Chinese Academy of Sciences, Wuhan 430072, China; 6 Institute of Marine Environment and Ecology and Center of Excellence for the Oceans, National Taiwan Ocean University, 2, Pei-Ning Road, Keelung 20224, Taiwan

**Keywords:** size diversity, biodiversity–ecosystem functioning, trophic transfer efficiency, functional diversity, body size, predator–prey dynamics

## Abstract

Body size exerts multiple effects on plankton food-web interactions. However, the influence of size structure on trophic transfer remains poorly quantified in the field. Here, we examine how the size diversity of prey (nano-microplankton) and predators (mesozooplankton) influence trophic transfer efficiency (using biomass ratio as a proxy) in natural marine ecosystems. Our results support previous studies on single trophic levels: transfer efficiency decreases with increasing prey size diversity and is enhanced with greater predator size diversity. We further show that communities with low nano-microplankton size diversity and high mesozooplankton size diversity tend to occur in warmer environments with low nutrient concentrations, thus promoting trophic transfer to higher trophic levels in those conditions. Moreover, we reveal an interactive effect of predator and prey size diversities: the positive effect of predator size diversity becomes influential when prey size diversity is high. Mechanistically, the negative effect of prey size diversity on trophic transfer may be explained by unicellular size-based metabolic constraints as well as trade-offs between growth and predation avoidance with size, whereas increasing predator size diversity may enhance diet niche partitioning and thus promote trophic transfer. These findings provide insights into size-based theories of ecosystem functioning, with implications for ecosystem predictive models.

## 1. Introduction

A major challenge in contemporary ecology is the development of a more mechanistic understanding of the relationships between biodiversity and ecosystem functioning (BEF) [1]. Although biodiversity has been demonstrated to promote efficiency of resource use and productivity within a trophic level, no consensus has been reached concerning the mechanism, with some advocating for sampling effects (i.e. likelihood of finding a species of high productivity) and others arguing for complementarity (i.e. niche partitioning and/or facilitation) [2]. Moreover, the issue of BEF becomes much more complicated when considering more than one trophic level. For example, experimental manipulation of insect and plant diversity has shown that resource diversity counteracts the positive effect of consumer diversity on the efficiency of resource use [3]. These studies point out apparently contrasting effects of predator and prey diversity on trophic transfer; nevertheless, studies on how the interaction of diversity across trophic levels influences measures of ecosystem function, such as trophic transfer efficiency (TTE) remain scarce [4]. Another knowledge gap is that most BEF research consists of manipulation studies in controlled environments. As Hillebrand & Cardinale [5] stress, manipulation studies are important for their control capacity but are still unrealistic (i.e. simplified interactions and too few species) and therefore, any conclusions are prone to bias. Thus, in order to complement and clarify our understanding of BEF, it is necessary to address these questions for natural communities.

A significant step towards understanding the role of biodiversity on ecosystem functioning has been made with the growing research on functional diversity, which focuses on functional traits rather than on species [6]. Recent studies on aquatic systems have found that functional-trait diversity tends to perform better than taxonomic diversity as a metric for linking community structure to aspects of ecosystem function such as carbon export and productivity [7]. In particular, individual size of plankton has been designated as the ‘meta-trait’ integrating several functional traits into one measurement [8,9]. The structuring role of body size in aquatic ecosystems arises because of physiological constraints as well as predator–prey mechanical and energetic constraints [10,11]. Physiological constraints emerge because metabolism scales with body size, and thus population traits such as abundance, secondary production and nutrient turnover rates, also scale with size [10]. With respect to predator–prey dynamics, predators tend to be larger than their prey [11], the size of predators generally conditions the size of their prey [12] and larger predators usually take advantage of a larger size range of prey [13].

Consequently, the size diversity of a community is expected to influence biomass transfer between trophic levels. This is a very old idea originally conceptualized in the seminal works of Elton [14] and Odum [15]. Unfortunately, this idea has not been fully explored, because most models have relied on Lindeman's [16] principles of trophic dynamics and assumed an average transfer efficiency of 10% (but see [17] for a derivation of TTE based on size-related predator–prey interactions). However, resurgent interest in size structure has recently revived the ideas of Elton [14] and Odum [15]. For example, Yvon-Durocher *et al.* [18] reported that the reduced cell sizes of prey (phytoplankton) accompanying warming in their mesocosm experiment increased trophic transfer (using the predator/prey biomass ratio as proxy). The authors hypothesized that enhanced prey turnover rates owing to metabolic acceleration and smaller cell size explained the increased energy flux. In another experiment, Steiner [19] explained that reduced predation effects with increasing phytoplankton cell size and diversity were related to reduced edibility, but only under conditions of nutrient enrichment. These findings demonstrate that prey (phytoplankton) size represents a pivotal trade-off between population growth rates and susceptibility to grazing by predators (zooplankton), meaning that larger cells experience slower growth, but also reduced mortality because their size exceeds handling capacity of predators and/or because they invest more energy in predation avoidance. From the perspective of predators, Ye *et al.* [20] analysed the size structure of mesozooplankton communities in the East China Sea (ECS), and found that zooplankton size diversity explained a significant proportion of the variation of the predator/prey biomass ratio. The authors proposed diet niche partitioning as the mechanism behind an observed positive effect of predator size diversity on trophic transfer.

To the best of our knowledge, no empirical study has focused on the concomitant effects of size diversity of predators and their potential prey on biomass trophic transfer. Thus, in this work, we explore the effect of the size diversity of predators (mesozooplankton excluding carnivores) and the size diversity of their potential prey (nano-microplankton) on biomass trophic transfer in natural planktonic communities. Here, size diversity is calculated as the analogue of the Shannon diversity index adapted to individual size distribution [21]. The ratio of mesozooplankton (predator) to nano-microplankton (prey) biomass in log-scale (log_10_(PPBR)) was used as a proxy for TTE from prey to predators [18,20]. TTE refers to the ecological efficiency of transferring biomass between adjacent trophic levels, thus the net result of integrating physiological and predator–prey dynamics. The choice of biomass ratio as a proxy is mainly due to the impossibility of routinely measuring turnover rates to estimate the ecological efficiency, which is the production rate (turnover rate × biomass). Based on the studies discussed above, we hypothesize that (i) a greater predator size diversity promotes trophic transfer and (ii) a greater prey size diversity hinders trophic transfer. We anticipate that the interactive effect of predator and prey size diversities has a stronger impact on trophic transfer than the effect of size diversity at a single trophic level. We also explored the effect of nutrients and temperature on size diversity and trophic transfer in order to detect environmental conditions favourable to trophic transfer, as well as to propose mechanisms underlying those relationships.

## 2. Material and methods

### (a) Sampling and sample processing

We collected 106 sets of samples from 11 cruises covering the ECS and waters east of Taiwan between May and October from 2009 to 2013 (appendix A in electronic supplementary material). Nano-microplankton were sampled with Go-Flo bottles at every 10-m depth interval from 10 m below the chlorophyll maximum depth to the surface, and cell size was measured with the FlowCAM [22]. Mesozooplankton were sampled with oblique tows of an Ocean Research Institute (ORI) net from 10 m above the sea floor (or from 200 m for deeper stations) to the surface, and organisms were analysed with the ZooSCAN [23]. Carnivores were removed from the mesozooplankton counts prior to data analyses. Mesozooplankton were sampled through the entire water column, because some of these organisms carry out a daily vertical migration but still feed in the photic zone. On average, 2000 and 3500 individuals were measured for mesozooplankton and nano-microplankton, respectively, in each sample. Sampling, sample preservation and sample digitizing procedures with the ZooSCAN and FlowCAM are detailed in appendix A, electronic supplementary material.

Sea surface temperature (SST), sea surface salinity (SSS), nitrate (NO_3_), phosphate (PO_4_) and silicate (SiO_3_) concentrations were determined according to standard methods [24]. Depth-integrated nutrient concentrations were calculated using multiple depth-specific measurements from above the mixed layer depth (MLD; see details in appendix A, electronic supplementary material).

### (b) Total biomass and plankton size diversity

Total biomass was estimated from individual biovolume using literature-based conversion factors for mesozooplankton and nano-microplankton (appendix B, electronic supplementary material). Size diversity was estimated from individual biovolume rather than biomass in order to retain the greatest possible accuracy of the two-dimensional size measurements. The major (M) and minor (m) axes of an ellipse containing the area of each individual were converted to ellipsoidal biovolume. We used the ellipsoidal volume (*EllipVol*) over the more popular equivalent spherical diameter (ESD), because most of the mesozooplankters in our samples were elongated. Nano-microplankton cell volumes were corrected for shrinkage owing to preservation. Size diversity (*μ*) corresponds to the analogue of the Shannon diversity index computed on the probability density function of individual biovolumes estimated with non-parametric kernel [21] (see appendix B, electronic supplementary material, for further details):
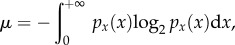
where *p_x_*(*x*) is the probability density function of size *x*, and *x* represents log(*EllipVol*). Here, individual biovolume was log-transformed to adjust the apparent variance of organisms of diverse size [25]. The calculation of size diversity is based on relative contribution and is therefore mathematically independent from total biomass; a statistical relationship between size diversity and total biomass would therefore be due to biological processes and not to mathematical artefact [20]. In our dataset, predator size diversity exhibits a significant negative correlation with prey biomass (*r* = −0.32, *p* < 0.0001), whereas prey size diversity exhibits a significant positive correlation with prey biomass (*r* = 0.29, *p* < 0.0001).

### (c) Trophic transfer efficiency

The ratio of mesozooplankton (predator) to nano-microplankton (prey) biomass in log-scale (log_10_(PPBR)) was used as a proxy for TTE from prey to predators [18,20]. In order to assess the suitability of the biomass ratio as a proxy for TTE calculated as the ratio of production rates (PPPR), we compared a parallel but reduced dataset (see appendix C, electronic supplementary material for detailed methodology). For the comparison, zooplankton biomasses and production rates were estimated only for copepods (representing 70–90% of total zooplankton biomass in our samples), and their growth rates were calculated using the ‘artificial cohort method’. Our comparison indicates that the proxy, log_10_(PPBR) and the direct TTE estimate, log_10_(PPPR), were strongly correlated (*r* = 0.84; *p* < 0.0001; *n* = 29; electronic supplementary material, figure C1). Furthermore, we tested how the potential uncertainty associated with using log_10_(PPBR) as a proxy for TTE could be propagated throughout our results. The uncertainty (e.g. standard errors of the log_10_(PPBR) : log_10_(PPPR)) was propagated via bootstrap, and the conclusions drawn from this study remained after accounting for this uncertainty (electronic supplementary material, figure C3).

### (d) Data analyses

We used linear mixed-effects modelling (LMM) to investigate which factors affect biomass TTE, with the trophic transfer proxy, log_10_(PPBR), as the response variable and predator and prey size diversities as well as environmental factors as explanatory variables. Pseudo-replication was accounted for by allowing the intercept to vary with sampling station as a random effect (i.e. 40 stations and thus 65 instead of 105 degrees of freedom in models with one explanatory variable). We then, investigated the extent of top-down and bottom-up control by exploring factors explaining predator and prey size diversity; that is, we considered the size diversity of prey or predators as the response variable, and the size diversity of predators or prey respectively, nutrient and temperature conditions as explanatory variables. For all models, we also report model fits with sea surface salinity as a way to dissociate temperature and nutrient effects from solely coastal–offshore differences. Furthermore, spatial autocorrelation of response variables was thoroughly explored and did not affect the conclusions of our study (electronic supplementary material, appendix D).

In each case, we first-ranked single explanatory variables using univariate models, and then tested all possible combinations of explanatory variables to determine the most parsimonious model according to the Akaike's information criterion corrected for sample size (AICc). In order to gain greater insights into potential mechanisms, the most parsimonious model explaining the predator (or prey) size diversity was identified through inclusion and exclusion of size diversity of prey (or predators) as one of the explanatory variables. Nutrient concentrations were log-transformed to approach normality prior to analyses. The basic statistics and pairwise relationships for all of the variables used in this study are presented in appendix E, electronic supplementary material.

Size diversity calculations and data analyses were carried out with Matlab^®^ v. 7.9 (The Mathworks, Inc., Natick, MA). Models were implemented with the ‘lme’ function in the nlme package [26] of R (R Development Core Team, 2010), and the most parsimonious models were selected with the ‘dredge’ function of the MuMIn package in R [27].

## 3. Results

**Figure 1.**
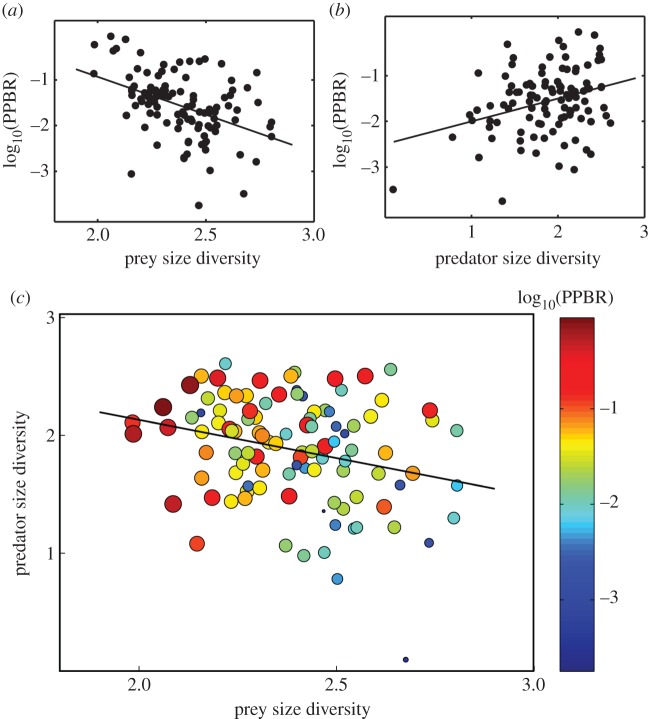
Effects of (*a*) prey size diversity on biomass transfer efficiency (log_10_(PPBR)) (*r* = −0.43, *p* < 0.0001), (*b*) predator size diversity on biomass transfer efficiency (log_10_(PPBR)) (*r* = 0.32, *p* < 0.0001) and (*c*) prey and predator size diversities on biomass transfer efficiency (log_10_(PPBR)). In panel (*c*), colour as well as symbol size indicate the biomass transfer efficiency. The solid line represents the relationship between prey and predator size diversity (*r* = −0.27, *p* = 0.006).

Mesozooplankton biomass was correlated with the biomass of potential prey, nano-microplankton, even though the relationship is not especially strong (*r* = 0.23; *p* = 0.02; electronic supplementary material, figure B2). The best single factor explaining prey–predator biomass transfer efficiency (log_10_(PPBR) as proxy) was the size diversity of prey (table 1). The most parsimonious model explaining biomass transfer was the interaction of predator and prey size diversity (table 1 and figure 1; electronic supplementary material, figure F1a and figure F2a). We also note that predator and prey size diversities exhibit a significant, albeit weak, negative correlation (table 2; *r* = −0.27; *p* = 0.006; *n* = 106).

**Table 1.** Results of LMM explaining the biomass transfer efficiency (log_10_(PPBR)). The best univariate explanatory variable is prey size diversity according to the Akaike information criterion corrected for sample size (AICc) and highlighted in italics. The most parsimonious model (with the lowest AICc) includes the interaction of predator and prey size diversities. Intrinsic variables (plankton) and extrinsic (environment) are separated by a division line. (*p < 0.05, **p < 0.005, ***p < 0.0005.)

response variable: log_10_(PPBR)						
		slope				
one explanatory variable	AICc	estimate	s.e.	*t*-value	*p*-value	d.f.
predator size diversity	221.03	0.497	0.145	3.423	0.001**	65
prey size diversity	*209.99*	−1.655	0.332	−4.980	<0.0001***	65
water salinity (SSS)	232.17	0.009	0.025	0.376	0.71	65
water temperature (SST)	225.68	0.046	0.018	2.614	0.011*	65
NO_3_	228.00	−0.147	0.071	−2.087	0.041*	65
PO_4_	227.95	−0.755	0.359	−2.098	0.040*	65
SiO_3_	223.86	−0.278	0.094	−2.955	0.004**	65
most parsimonious model: log_10_(PPBR) ∼ prey size diversity × predator size diversity AICc: 205.63.

**Table 2.** Results of LMM explaining (*a*) predator size diversity and (*b*) prey size diversity. The best model (i.e. predator size diversity explained by SiO_3_; prey size diversity explained by temperature) is selected according to the Akaike information criterion corrected for sample size (AICc) and highlighted in italics. The most parsimonious model explaining the predator (or prey) size diversity was computed through including and excluding the size diversity of prey (or predators) as one of the explanatory variables. (*p < 0.05, **p < 0.005, ***p < 0.0005.)

		slope				
one explanatory variable	AICc	estimate	s.e.	*t*-value	*p*-value	d.f.
(*a*) response variable: predator size diversity						
prey size diversity	129.03	−0.648	0.226	−2.857	0.005**	65
water salinity (SSS)	130.63	0.040	0.015	2.560	0.01*	65
water temperature (SST)	128.44	0.033	0.011	2.962	0.004**	65
NO_3_	123.97	−0.161	0.043	−3.698	<0.0001***	65
PO_4_	128.36	−0.670	0.225	−2.979	0.004**	65
SiO_3_	*107.75*	−0.313	0.054	−5.760	<0.0001***	65
most parsimonious model: predator size diversity ∼ SiO_3_ + prey size diversity AICc: 107.44 excluding prey size diversity: predator size diversity ∼ SiO_3_ AICc: 107.75						
(*b*) response variable: prey size diversity						
predator size diversity	−59.36	−0.109	0.039	−2.818	0.006**	65
water salinity (SSS)	−51.83	−0.003	0.006	−0.515	0.61	65
water temperature (SST)	*−70.35*	−0.020	0.004	−4.491	<0.0001***	65
NO_3_	−58.11	0.047	0.018	2.574	0.01*	65
PO_4_	−52.57	0.095	0.096	0.997	0.32	65
SiO_3_	−60.73	0.075	0.024	3.066	0.003**	65
most parsimonious model: prey size diversity ∼ SST + pred. size diversity AICc: −71.45 excluding predator size diversity: prey size diversity ∼ SST + SiO_3_ + PO_4_ + SST × SiO_3_ AICc: −71.34						

**Figure 2.**
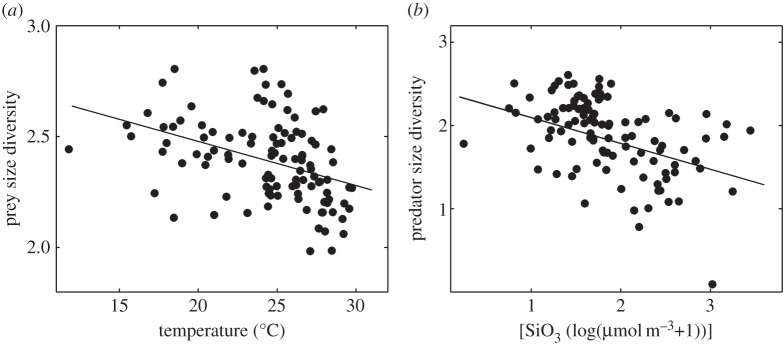
Effects of (*a*) temperature on prey size diversity (*r* = −0.40, *p* < 0.0001) and (*b*) silicate concentration on predator size diversity (*r* = −0.49, *p* < 0.0001).

The concentration of SiO_3_ was the best single factor explaining predator size diversity (table 2*a* and figure 2*b*), whereas temperature was the best single factor explaining prey size diversity (table 2*b* and figure 2*a*). Predator size diversity tended to be lower in waters with high SiO_3_ concentrations, and prey size diversity tended to be lower in warm waters. The most parsimonious model explaining predator size diversity included prey size diversity as the second factor accompanying the best single predictor, as did the model for prey size diversity (table 2; see ranking of all possible models in electronic supplementary material, figure F1b and figure F1c). We also considered the most parsimonious models for predator size diversity in the absence of prey size diversity and for prey size diversity in the absence of predator size diversity as explanatory variables. Under these model constraints, the most parsimonious model explaining predator size diversity included only SiO_3_, and that explaining prey size diversity included the effects of PO_4_ and SiO_3_ in addition to that of temperature (table 2; electronic supplementary material: second rows of figure F1c and figure F1b, and first row of figure F2d).

In summary, TTE (log_10_(PPBR)) exhibited a significant positive correlation with temperature and significant negative correlations with nutrient concentrations, associated with a tendency for prey size diversity to be low and predator size diversity to be high in those conditions (tables 1 and 2; figures 1 and 2). High TTE was primarily related to low prey size diversity. Yet, predator size diversity exerted additional secondary positive effects on TTE, especially when size diversity of prey was high (figure 1*c*). In accordance, the most parsimonious model to explain TTE is the interaction of prey and predator size diversities (table 1).

## 4. Discussion

### (a) Predator and prey size diversity affect trophic transfer efficiency

We found that both predator and prey size diversity exhibit significant relationships with TTE in planktonic communities (table 1 and figure 1*c*). While this is solely based on correlation analysis, our findings support both hypotheses: (i) predator size diversity promotes biomass trophic transfer through enhanced diet niche partitioning [20] and (ii) prey size diversity hinders biomass transfer via an increasing incidence of predation defence [19] or a slowing of population turnover rates with increasing cell size [18]. To the best of our knowledge, this is the first field study showing the opposite effect of predator and prey size diversity on biomass transfer efficiency. In this study, the size diversity of prey has a greater effect on trophic transfer than the size diversity of predators. Yet, the interaction of predator and prey size diversities is significantly more important than the size diversity of either predator or prey alone (table 1 and electronic supplementary material, figure F1a). Specifically, when the size diversity of prey is low, trophic transfer tends to be high; when the size diversity of prey is high, trophic transfer tends to be higher with high predator size diversity (figure 1*c*). We hypothesize that enhanced diet niche partitioning related to high predator size diversity would mitigate the negative effects of prey diversity. Another alternative and non-exclusive hypothesis would be that under conditions of low prey size diversity, the mesozooplankton with a target size range of prey that does not overlap the size of available prey may relax their size-based selectivity, thus increasing the target size range of prey. Wider target size ranges among the mesozooplankton could on the one hand potentially enhance competition for the same prey, whereas, on the other hand, potentially decrease prey control via lower than expected ingestion rates from such a diverse mesozooplankton community. This idea has been developed through a mechanistic model in which size dependency of prey selection is divided into a fixed mechanical ingestion dependency, and a behavioural selection dependency driven by trade-offs related to food availability [28].

One might argue that the promotion of trophic transfer by predator size diversity may be solely attributed to an accompanying increase in the proportion of large (but rare) predators. This greater proportion of large predators would increase the predator–prey individual mass ratio (PPMR), enhance attack rates, decrease handling times and ultimately promote predation success [29]. Yet, according to our further analyses (electronic supplementary material, appendix G), the effect of PPMR on log_10_(PPBR) was weaker than that of the ratio of predator–prey size diversities. Likewise, the effect of average body size on log_10_(PPBR) was weaker than the effect of size diversity (electronic supplementary material, table G1) and supports our hypothesis.

The interaction of bitrophic diversities as ultimately determining predation performance has recently been reported in experimental studies [30,31]. Using mesocosm manipulations of prey and predator species richness, both Gamfeldt *et al.* [31] and Saleem *et al.* [30] highlighted the importance of the presence of multiple, functionally distinct species for determining the strength of predation performance. The role of resource partitioning (via specialization) as a mechanism for reducing total prey (resource) standing stock and for increasing total consumer biomass seems to be generalizable across natural communities, as reviewed by Duffy *et al.* [32] and reported in a meta-analysis [33]. The underlying mechanism proposed in these studies is similar to that reflected in our results, albeit we focus on size diversity rather than on taxonomic diversity. Neither the Saleem *et al.* [30] nor Gamfeldt *et al.* [31] experiments identified the negative effect of prey diversity on predation found in our study. This may be due to the low experimental diversity levels (max. three to five species) and because none of the manipulated prey species exhibited predation defences, a mechanism likely to be common in natural communities. In addition, these experiments used species richness as a measure of diversity, whereas we measured the entropy of individual sizes, which may be better suited for characterizing functional differentiation especially given the identification of size as a ‘meta-trait’ [7–9].

### (b) Trophic transfer efficiency in the environmental context

We found that trophic transfer exhibits significant negative relationships with nutrient concentrations, and a significant positive relationship with temperature (table 1). This agrees with previous findings suggesting that trophic transfer increases with food scarcity [34–36]. Indeed, Calbet's [36] global comparative study on mesozooplankton ingestion rates concluded that the relative proportion of primary production removed by mesozooplankton decreased with nutrient availability. Moreover, recent estimates of mesopelagic fish biomass have indicated a 10-fold greater biomass than has been traditionally estimated by models, suggesting underestimation of trophic transfer from primary producers to fishes in the oligotrophic ocean [37]. Those high transfer efficiencies in oligotrophic conditions have been explained as owing to the fast turnover rates of the dominant small primary producers and therefore, an enhanced capacity to support more predator biomass as well as a more stable and clear water column that also aids greater predator–prey coupling [34–36]. Our results provide further insights, by suggesting that prey size diversity may tend to be low and predator size diversity to be high in the stable oligotrophic environments (table 2), and therefore facilitate a greater efficiency of trophic transfer (figure 1).

### (c) Relationship between predator and prey size diversities

Another interesting finding is a significant, although weak, negative correlation between predator and prey size diversities (figure 1*c*). This finding contradicts a positive relationship of diversities (generally measured as species richness) between trophic groups reported by previous studies in terrestrial systems [38,39]. Yet, in the case of planktonic systems, the relationship between predator and prey diversities has already been reported to be weaker and with a variable sign [40,41]. This contrast may be partly explained by the knowledge that increasing terrestrial plant diversity is often accompanied by the creation of new spatial niches for herbivores, whereas in the water column, increased habitat complexity accompanying diversity of primary producers does not necessarily occur, because phytoplankton are unicellular. Besides, the positive effect of prey diversity on predator diversity via enhanced niche partitioning might be overcome in planktonic ecosystems because of a greater incidence of prey defence mechanisms accompanying increasing prey diversity [5]. Lastly, we cannot rule out the possibility that the negative correlation between predator and prey size diversities found in our study might be due to differences in the major forces driving them. While predator size diversity is mainly affected by food availability effects on secondary production in the ECS [42], prey size diversity is mainly driven by temperature via metabolic constraints [18] (table 2).

### (d) Future research

Omnivory and intraguild predation have been reported to affect community size structure [43] and ecosystem function [44]. Although we did go to considerable effort to remove carnivores from the predators, we do acknowledge that our methodology limited our ability to account for these interactions. Calbet & Landry [45] demonstrated that the proportion of microzooplankton grazing on phytoplankton is relatively constant (60–75%) across environments; nevertheless, they also suggest that perhaps the length of protistan predatory chains may also play a role in TTE. Mesocosm manipulations further suggest that mesozooplankton may switch from phytoplankton to grazing on ciliates in warm nutrient-limited conditions [46]. Thus, we encourage future studies to explore and account for the effect of intraguild predation within mesozooplankton and microplankton groups when analysing TTE among these plankton groups.

## 5. Final remark

This study is, to the best of our knowledge, the first to report that the synergistic effect of size diversity at two contiguous trophic levels is stronger for biomass trophic transfer than the effect of size diversity at a single trophic level. While the size diversity of prey hinders transfer to consumers in planktonic communities, the size diversity of predators may promote the efficiency of resource utilization. Size-based top-down control seems to occur mainly via niche partitioning, whereas size-based bottom-up control takes place mainly via size-based metabolic constraints of unicellular growth and the trade-off between growth and predation avoidance. Furthermore, trophic transfer would be stronger in more oligotrophic, warm waters. Our findings rely on correlation analyses and cannot represent proof of causation; however, they do provide insights into size-based theories of ecosystem functioning and suggest mechanisms to test for modelling predictions of fisheries yields as well as the response of plankton size structure to global warming: a factor critical to the functioning of pelagic ecosystems.

## Supplementary Material

GarciaComasetal_SupplementaryMaterial_AppendicesAtoG_ProceedingsB

## Data accessibility

Dryad data reference: http://dx.doi.org/10.5061/dryad.300k7.
