# Supplementary material for: Prey size diversity hinders biomass trophic transfer and predator size diversity promotes it in planktonic communities
Source: Proc Biol Sci. 2016 Feb 10;283(1824):20152129. doi: 10.1098/rspb.2015.2129 (PMC4760158; doi:10.1098/rspb.2015.2129)
Supplement: GarciaComasetal_SupplementaryMaterial_AppendicesAtoG_ProceedingsB [file rspb20152129supp1.docx]

**Prey size diversity hinders biomass trophic transfer**

**and predator size diversity promotes it in planktonic communities**

C. García-Comas, A. R. Sastri, L. Ye, C.-Y. Chang, F.-S. Lin, M.-S. Su, G.-C. Gong

and C.-h. Hsieh

**SUPPLEMENTARY MATERIAL**

**Appendix A: Sampling and sample processing**


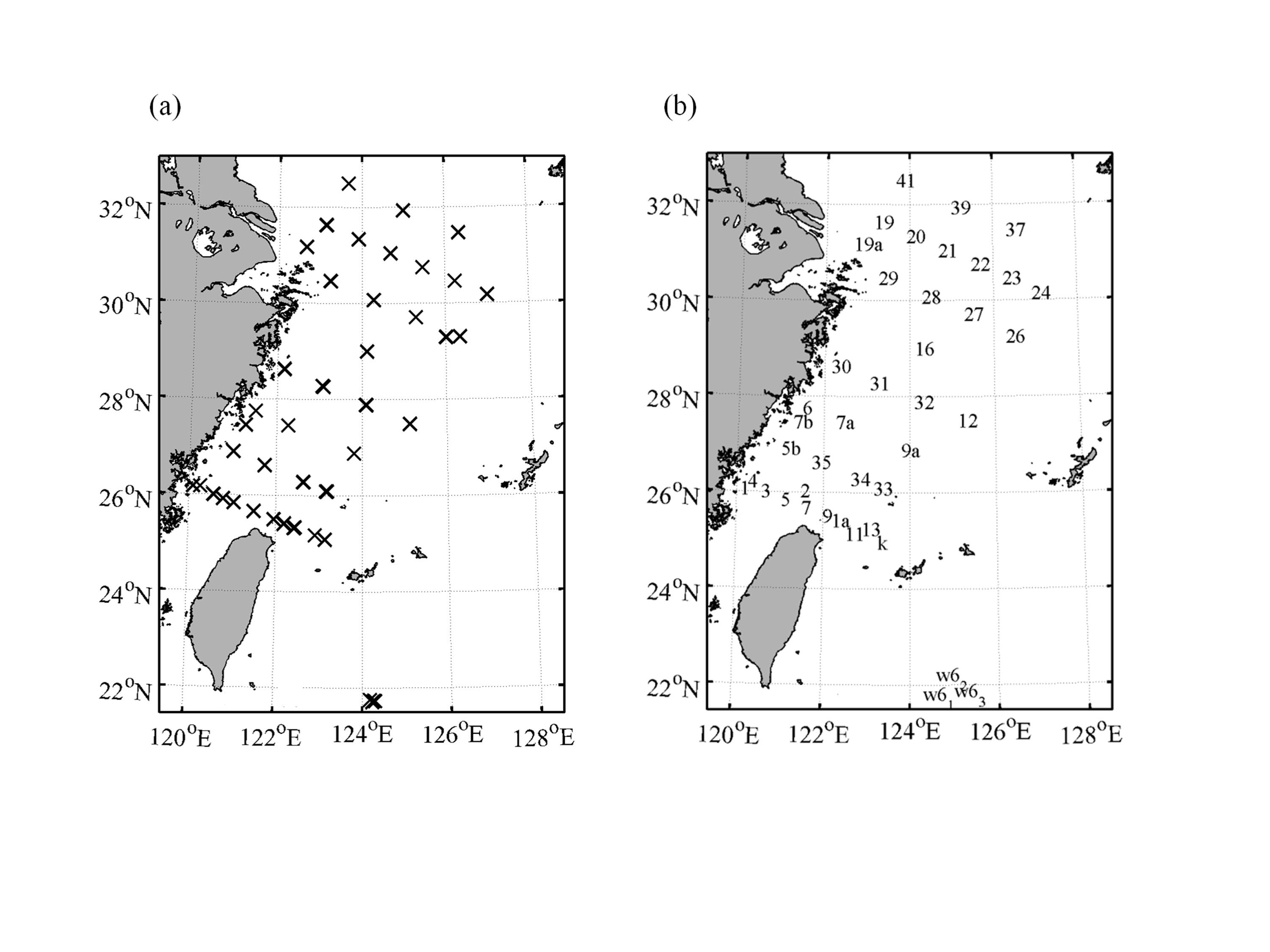


Figure A1. (a) Sampling locations in the East China Sea and waters east of Taiwan and (b) names of stations (position adjusted slightly for readability).

Table A1. Grey-filled table entries represent stations (out of the 40 stations presented in Fig. A1) sampled on each of 11 cruises. A total of 106 samples were analyzed in this study.

| Stations/  Cruises | May  2009 | July  2009 | April  2010 | May  2010 | July  2010 | Sept.  2010 | July  2011 | Aug.  2011 | Oct.  2011 | July  2012 | May  2013 |
| --- | --- | --- | --- | --- | --- | --- | --- | --- | --- | --- | --- |
| 1 |  |  |  |  |  |  |  |  |  |  |  |
| 1a |  |  |  |  |  |  |  |  |  |  |  |
| 3 |  |  |  |  |  |  |  |  |  |  |  |
| 4 |  |  |  |  |  |  |  |  |  |  |  |
| 5 |  |  |  |  |  |  |  |  |  |  |  |
| 5b |  |  |  |  |  |  |  |  |  |  |  |
| 6 |  |  |  |  |  |  |  |  |  |  |  |
| 7 |  |  |  |  |  |  |  |  |  |  |  |
| 7a |  |  |  |  |  |  |  |  |  |  |  |
| 7b |  |  |  |  |  |  |  |  |  |  |  |
| 9 |  |  |  |  |  |  |  |  |  |  |  |
| 9a |  |  |  |  |  |  |  |  |  |  |  |
| 11 |  |  |  |  |  |  |  |  |  |  |  |
| 12 |  |  |  |  |  |  |  |  |  |  |  |
| 13 |  |  |  |  |  |  |  |  |  |  |  |
| 16 |  |  |  |  |  |  |  |  |  |  |  |
| 19 |  |  |  |  |  |  |  |  |  |  |  |
| 19a |  |  |  |  |  |  |  |  |  |  |  |
| 20 |  |  |  |  |  |  |  |  |  |  |  |
| 21 |  |  |  |  |  |  |  |  |  |  |  |
| 22 |  |  |  |  |  |  |  |  |  |  |  |
| 23 |  |  |  |  |  |  |  |  |  |  |  |
| 24 |  |  |  |  |  |  |  |  |  |  |  |
| 26 |  |  |  |  |  |  |  |  |  |  |  |
| 27 |  |  |  |  |  |  |  |  |  |  |  |
| 28 |  |  |  |  |  |  |  |  |  |  |  |
| 29 |  |  |  |  |  |  |  |  |  |  |  |
| 30 |  |  |  |  |  |  |  |  |  |  |  |
| 31 |  |  |  |  |  |  |  |  |  |  |  |
| 32 |  |  |  |  |  |  |  |  |  |  |  |
| 33 |  |  |  |  |  |  |  |  |  |  |  |
| 34 |  |  |  |  |  |  |  |  |  |  |  |
| 35 |  |  |  |  |  |  |  |  |  |  |  |
| 37 |  |  |  |  |  |  |  |  |  |  |  |
| 39 |  |  |  |  |  |  |  |  |  |  |  |
| 41 |  |  |  |  |  |  |  |  |  |  |  |
| k |  |  |  |  |  |  |  |  |  |  |  |
| w6_1_ |  |  |  |  |  |  |  |  |  |  |  |
| w6_2_ |  |  |  |  |  |  |  |  |  |  |  |
| w6_3_ |  |  |  |  |  |  |  |  |  |  |  |

Zooplankton were sampled with an ORI (Ocean Research Institute) net (330 µm mesh size; 1.6 m mouth diameter). Nets were towed obliquely at 0.3 m s^-1^ from 10 m above the bottom to the surface. Upon retrieval of the net, the cod-end contents were immediately preserved and stored in 4% buffered formalin until analysis with the ZooSCAN. In the laboratory, each sample was first rinsed with filtered water and then split until the sub-sample was sufficiently dilute to limit contact between organisms on the ZooSCAN 15x24 cm scanning frame (~2000 counts). This subsample was then scanned at 2400 dpi (pixel resolution of 10.58 μm2) and analyzed with the Zooprocess Integrated System [1].

Nano-microplankton were sampled with Go-Flo bottles at 10 meter (m) depth intervals extending from 10 m below the depth of the chlorophyll maximum to the surface. An equivalent sub-sample volume from each depth was mixed to yield two 1-liter (L) samples (each representing an integrated water column sample). One sample was fixed with 0.2% paraformaldehyde for analysis of small cells (2-50 μm ESD), and the other with 2% Lugol’s solution for analysis of larger cells (50-200 μm ESD). Fixed samples were refrigerated until analysis. Back in the lab, the Lugol-fixed samples were gently mixed, and then sub-sampled twice (100 ml each). Each aliquot was placed in a sedimentation chamber for 24h in order to concentrate large cells. The concentrated cells were recovered in a small volume, diluted with filtered water up to 4mL, and finally analyzed using the FlowCAM [2]. To correctly measure large cells, we set the FlowCAM on auto-image mode with a flow cell FC300 (equivalent to a 4x objective) and speed of Fast4. For the paraformaldehyde-fixed sample, we collected 3 aliquots, of 2 to 5 mL each depending on the concentration (minimum concentration matching ~1500 counts with the FlowCAM). Aliquots (replica) were then analyzed with the FlowCAM on auto-image mode with FC100 (equivalent to x10-x20 objectives) and Fast8 speed. On average, the 2 replica of concentrated large cells recorded ~600 counts each, while the other 3 replica had ~3000 counts.

Sea surface temperature (SST) and salinity (SSS) were recorded with a SeaBird CTD profiler (SBE9/11 plus, SeaBird Inc., USA). Nitrate (NO_3_), phosphate (PO_4_) and silicate (SiO_3_) concentrations were measured from water samples collected at discrete depths (e.g., 0, 10, 25, 50, 75, 100 m depth) with Go-Flo bottles, and stored in liquid nitrogen until analysis. Analytical methods are described by Gong *et al.* [3]. We calculated their depth-weighted integrated values measured above the mixed layer depth (MLD) following the Levitus thermal criteria (i.e., the depth at which ΔT with SST was 0.5°C; [4]).

References:

1. Gorsky, G., Ohman, M.D., Picheral, M., Gasparini, S., Stemmann, L., Romagnan, J.-B., Cawood, A., Pesant, S., García-Comas, C., Prejger, F. 2010 Digital zooplankton image analysis using the ZooScan integrated system. *J. Plankton Res.* **32**, 285-303. (doi:10.1093/plankt/fbp124).

2. Sieracki, C.K., Sieracki, M.E., Yentsch, C.S. 1998 An imaging-in-flow system for automated analysis of marine microplankton. *Mar. Ecol. Progr. Ser*.**168**, 285–296. (doi:10.3354/meps168285).

3. Gong, G.-C., Shiah, F.-K., Liu, K.-K., Wen, Y.-H., Liang, M.-H. 2000 Spatial and temporal variation of chlorophyll a, primary productivity and chemical hydrography in the southern East China Sea. *Cont. Shelf Res.* **20**, 411-436. (doi:10.1016/S0278-4343(99)00079-5).

4. Levitus, S.1982 Climatological Atlas of the World Ocean, NOAA/ERL GFDL Professional Paper 13, Princeton, N.J., 173 pp. (NTIS PB83-184093).

**Appendix B:** **Calculation of size diversity and total biomass using FlowCAM and ZooSCAN measurements**

*Individual biovolume:*

Individual plankton biovolume was computed from measurements made with the ZooSCAN (mesozooplankton: predators) and FlowCAM (nano-microplankton: potential prey). The major (*M*) and minor (*m*) axes of a perfect ellipse containing the area of each individual were measured and used to calculate the ellipsoidal biovolume (*EllipVol*):

$$\text{E}llipVol=\left(4/3\right)\pi\left(M/2\right)\left(m/2\right)^{2}$$

In the case of nano-microplankton, we corrected the estimated volume for shrinkage due to preservation according to Montagnes (1994):

$$cellVol=1.33\text{ estimated cell volume}$$

*Kernel density estimates:*

Detrital objects were detected, by examining the individual thumbnail images obtained with the FlowCAM and ZooSCAN, and discarded prior to computing the probability density function (pdf) of plankton biovolume. Likewise, carnivores were discarded from the mesozooplankton. Following Schartau *et al.* [1], individual biovolume values were log-transformed. Mesozooplankton kernel density was estimated following the non-parametric estimate of optimal bandwidth proposed by Botev *et al.* [2]. In the case of nano-microplankton, the volume of subsample replicates was variable, so we could not directly compute the pdf on individual biovolumes. Instead, we arranged individual biovolumes for each subsample in size spectra normalized by the sampled volume. Then we added the average of spectra for the three 0-50 ml subsample replicates to the average spectra of the two 50-200 ml subsample replicates. The spectra had 100 bins of same width in logarithmic scale and ranged from 2-μm to 200-μm ESD equivalent ellipsoidal volume. The pdf was computed on the basis of a normal kernel function estimated for the 100 bins (see examples in Fig. B1).

*
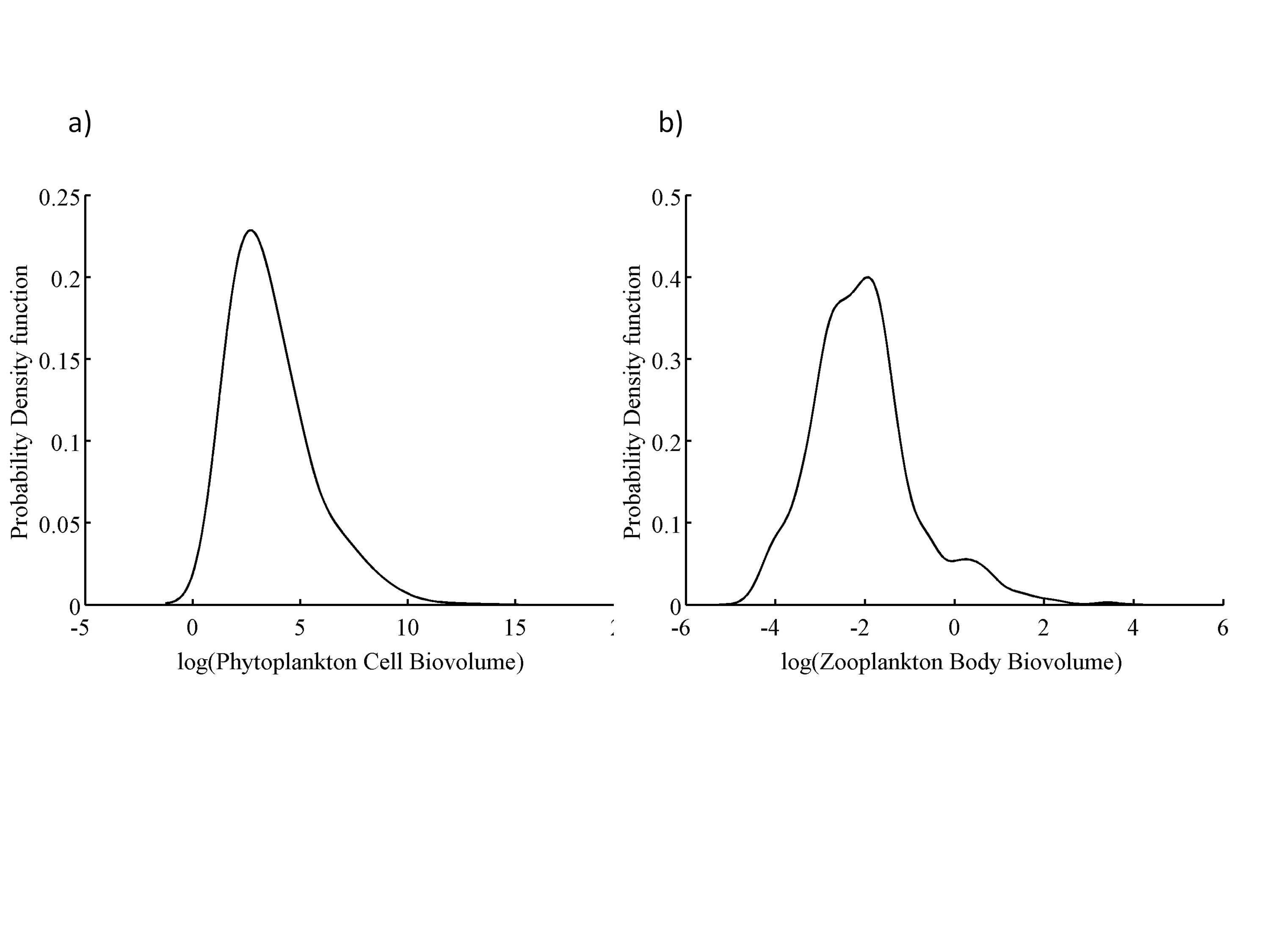
*

Figure B1. Example of probability density functions of (a) nano-microplankton log(individual μm^3^) and (b) mesozooplankton log(individual mm^3^).

*Size diversity:*

Size diversity (μ) corresponds to the equivalent continuous version of the Shannon-Wiener index traditionally used for calculations of species diversity [3]. This index is based on information theory and measures the entropy of the distribution:

$$\mu=\int_{0}^{+\infty} p_{x}(x)\log_{2}p_{x}(x)dx$$

where, p_x_(x) is the probability density function of size x, being x log(EllipVol). We chose not to standardize the size diversity by the geometric mean of individual sizes of a community because our samples were collected using consistent devices (see justification in [4]).

*Total biomass:*

Individual biomass was computed from biovolume following available literature-based conversions [5-9]. Conversion factors for nano-microplankton are presented in Table B1. In the case of mesozooplankton, we used conversion factors calculated by Alcaraz *et al.* [5] from images of zooplankton in the western Mediterranean (Table B2).

Table B1. Conversion factors for nano-microplankton cell volume to carbon content.

| ESD | Cell type | Equation  C(pg) & V(µm ^3^) | Reference |
| --- | --- | --- | --- |
| <20 µm | nanoplankton | logC=-0.583+0.860logV | Menden-Deuer and Lessard 2000 |
| 20-50 µm | Protists | logC=-0.665+0.939logV | Menden-Deuer and Lessard 2000 |
| >50 µm | Dinoflagellates | logC=-0.353+0.864logV | Menden-Deuer and Lessard,2000 |
| >50 µm | Diatoms | logC=-0.933+0.881logV | Menden-Deuer and Lessard 2000 |
| >50 µm | Naked Ciliates | logC=-0.639+0.984logV | Putt and Stoecker 1989 |
| >50 µm | Tintinnid Ciliates | logC=-0.168+0.841logV | Verity and Langdon 1984 |
| >50 µm | Other | logC=-0.665+0.939logV | Menden-Deuer and Lessard 2000 |

Table B2. Conversion factors for mesozooplankton body length to carbon content.

| Length (M) | Equation  C(mg) & V(mm ^3^) |
| --- | --- |
| <5mm | C=0.0825+0.0780V |
| ≥5mm | C=0.0514+0.0515V |

The sum of individual biomass counted from sub-samples using the FlowCAM and ZooSCAN was finally expressed in common units of mg C /mm^3^. The relationship of nano-microplankton and mesozooplankton biomass was significantly positive, yet weak (Fig. B2).


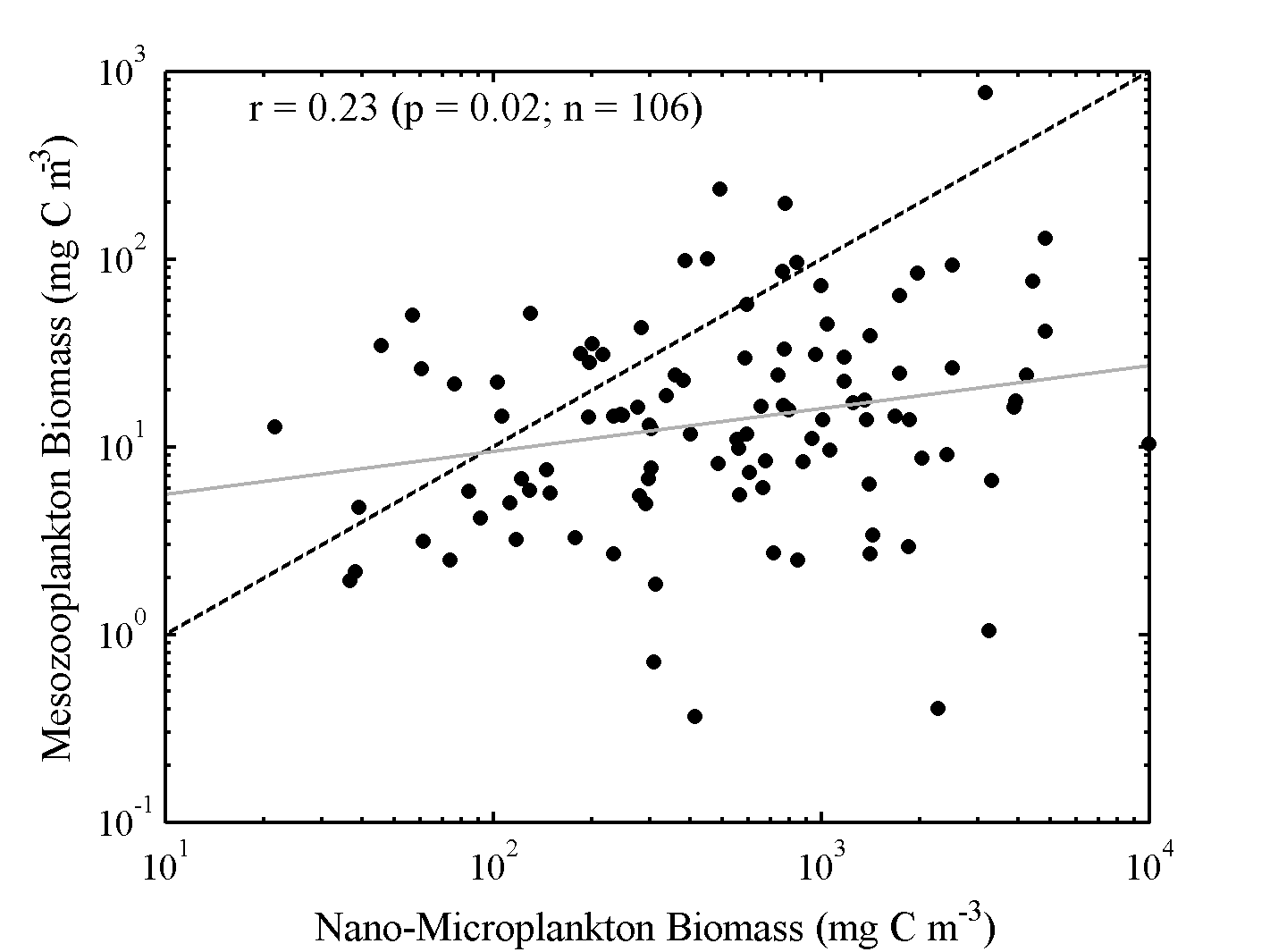


Figure B2. Log-scale relationship between predator (i.e., mesozooplankton) biomass and the biomass of its potential prey (i.e., nano-microplankton); the solid line represents the best-fit regression line. The dashed line corresponds to the 10% average biomass transfer efficiency generally assumed in models following Lindeman (1942).

References:

1. Schartau, M., Landry, M.R., Armstrong, R.A. 2010 Density estimation of plankton size spectra: a reanalysis of IronEx II data. *J. Plankton Res.* **32**, 1167-1184. (doi:10.1093/plankt/fbq072).

2. Botev, Z.I., Grotowski, J.F., Kroese, D.P. 2010 Kernel density estimation via diffusion. *Annals of Statistics* **38**: 2916–2957. (doi:0.1214/10-AOS799).

3. Quintana, X.D., Brucet, S., Boix, D., Lopez-Flores, R., Gascon, S., Badosa, A., Sala, J., Moreno-Amich, R., Egozcue, J.J. 2008 A nonparametric method for the measurement of size diversity with emphasis on data standardization. *Limnol. Oceanogr. Methods* **6**, 75-86. (doi:10.4319/lom.2008.6.75).

4. Ye, L., Chang, C.-Y., García-Comas, C., Gong, G.-C., Hsieh, C.-h. 2013 Increasing zooplankton size diversity enhances the strength of top-down control on phytoplankton through diet niche partitioning. *J. Anim. Ecol.* **82**, 1052-1061. (doi:10.1111/1365-2656.12067).

5. Alcaraz, M., Saiz, E., Calbet, A., Trepat, I., Broglio, E. 2003 Estimating zooplankton biomass through image analysis. *Mar. Biol.* **143**, 307-315. (doi:10.1007/s00227-003-1094-8).

6. Menden‐Deuer, S., Lessard, E.J. 2000 Carbon to volume relationships for dinoflagellates, diatoms, and other protist plankton. *Limnol. Oceanogr.* **45**, 569‐579. (doi:10.4319/lo.2000.45.3.0569).

7. Montagnes, D.J.S., Berges, J.A., Harrison, P.J., Taylor, F.J.R. 1994 Estimating Carbon, Nitrogen, Protein, and Chlorophyll a from Volume in Marine Phytoplankton. *Limnol. Oceanogr.* **39**, 1044‐1060. (doi:10.4319/lo.1994.39.5.1044).

8. Putt, M., Stoecker, D.K. 1989. An experimentally determined carbon: volume ratio for marine "oligotrichous" ciliates from estuarine and coastal waters. *Limnol. Oceanogr*. **34**, 1097‐1103. (doi:10.4319/lo.1989.34.6.1097).

9. Verity, P.G., Langdon, C. 1984 Relationships between Lorica volume, carbon, nitrogen, and ATP content of tintinnids in Narrangansett Bay. *J. Plankton Res.* **6**, 859‐868. (doi:10.1093/plankt/6.5.859).**Appendix C: Justification for using biomass ratio as a proxy of transfer efficiency in plankton**

Trophic transfer efficiency (TTE) in plankton is defined as:

$\frac{{Production}_{ZP}}{{Production}_{PHY}}=\frac{{Growth}_{ZP}\times{Biomass}_{ZP}}{{Growth}_{PHY}\times{Biomass}_{PHY}}$,

where *Production_ZP_* and *Production_PHY_* are the production of zooplankton and phytoplankton, *Growth_ZP_* and *Growth_PHY_* are the weight-specific growth rates of zooplankton and phytoplankton, and *Biomass_ZP_* and *Biomass_PHY_* are the biomass of zooplankton and phytoplankton, respectively. However, production ratio (TTE) has rarely been measured, because measuring production rates of zooplankton and phytoplankton simultaneously is difficult and time consuming. In planktonic systems, the variation of TTE is to a large extent determined by the variation of $\frac{{Biomass}_{ZP}}{{Biomass}_{PHY}}$ (i.e. the biomass ratio) since biomass is much more variable than specific growth rates [1]. As a consequence, the biomass ratio is often used as a proxy for TTE (e.g. [2-4]). This is the rationale for using the biomass ratio as a proxy for TTE in the current study.

To further justify the use of this proxy for our study area, we carried out shipboard incubation experiments at *in situ* thermal conditions to simultaneously measure the production rate-based TTE and the biomass ratio of plankton communities in the East China Sea (and partly the South China Sea). Due to logistical challenges, we have obtained only 29 data points to date (Fig. C1a). Also, for zooplankton, we have focused only on copepods, because on average, copepods represent 70% to 90% of total zooplankton biomass, and the methods for measuring copepod growth rates are more widely accepted relative to other zooplankton groups. Here, copepod community weight-specific growth rates were measured using the Artificial Cohort Method as detailed by Lin *et al.* [5]. Individual copepod biomass (carbon) values were estimated from body size measurements and counts of individuals sampled with NorPac plankton nets (50 μm mesh, with a ring diameter of 45 cm) and then integrated through the water column. Copepod production rates were then estimated as the product of copepod growth rate and copepod biomass for all incubated size-classes. While, phytoplankton productivity and phytoplankton chlorophyll *a* concentration integrated through the euphotic zone were estimated using ^14^C assimilation and fluorometric methods, respectively [6]. Chlorophyll *a* concentration was transformed to carbon biomass with a conversion factor of 30 [7]. We then analyzed the relationship between the copepod/phytoplankton production ratio (log_10_(PPPR)) and copepod/phytoplankton biomass ratio (log_10_(PPBR)) using a univariate regression analysis. Our results indicate a positive and highly significant relationship between the production ratio and the biomass ratio (Fig. C1b). While our measurements are limited to copepods and not to all zooplankton, our findings do, to some extent, justify the use of the biomass ratio as a proxy for TTE in plankton systems, at least in the East China Sea.

Finally, we propagated the uncertainty of using log_10_(PPBR) instead of log_10_(PPPR) as proxy for TTE as a sensitivity test of the main results in this study. The error (σ) of the intercept and slope of the log_10_(PPBR):log_10_(PPPR) relationship was estimated via 1000 bootstraps of the studentized residuals [8]. The outputs of log_10_(PPPR)~size diversities were calculated for 1000 bootstraps of the estimated log_10_(PPPR) ± σ. Results of this sensitivity analysis were consistent with the results reported in this study using log_10_ PPBR) (Fig. C2; Fig. C3). However, notice that this exercise is just an approximation, as our measurements are limited to copepods and not to all zooplankton.

One may speculate why the biomass ratio (a ratio of static variables) can be a reasonable proxy for TTE (a ratio of rate variables). For example, consider a simple Lotka-Volterra system with a limit cycle. This model will give different ratios of prey and predator biomass depending on when we look at it. However, one potential explanation will be that the coupling-uncoupling of biomasses would be related to a certain extent to the size structure of both predator and prey at each state. The integration of those momentums would give the prevalent biomass ratio in a certain area, yet this is not fixed, it is dynamic and related to the environmental conditions (e.g., contrasting conditions of upwelling versus oligotrophy). There is certainly a need to further study this topic and determine when and why the biomass ratio can or cannot be a proxy for TTE. Nevertheless, this is beyond the scope of this study.

Table C1. Range and coefficient of variation (CV) of plankton and environmental variables, and their pairwise Pearson correlations (p≤0.0001***, p≤0.01**, p≤0.05*).

|  | Range | CV | C. B. | C. G. | C. P. | P. B. | P. G. | P. P. | T. | [NO3] | [PO4] | [SiO3] |
| --- | --- | --- | --- | --- | --- | --- | --- | --- | --- | --- | --- | --- |
| Cop. B.  (mgC m^-3^) | 0.05-7.3 | 1.54 | 1 | 0.21 | 0.96*** | 0.08 | 0.49** | 0.37* | -0.07 | 0.38* | 0.28 | 0.51** |
| Cop. Growth rate  (day^-1^) | 0.11-0.7 | 0.42 |  | 1 | 0.33 | 0.30 | 0.11 | 0.34 | -0.04 | 0.01 | 0.01 | -0.05 |
| Cop. Prod.  (mgC m^-3^ day^-1^) | 0.01-3.5 | 1.86 |  |  | 1 | 0.06 | 0.51** | 0.34 | -0.14 | 0.40* | 0.28 | 0.51** |
| Phy. B.  (mgC m^-3^) | 4.45-129.3 | 0.93 |  |  |  | 1 | -0.31 | 0.69*** | 0.13 | 0.34 | 0.25 | 0.29 |
| Phy. Growth rate  (day^-1^) | 0.09-2.6 | 0.69 |  |  |  |  | 1 | 0.28 | 0.02 | 0.07 | 0.002 | 0.17 |
| Phy. Prod.  (mgC m^-3^ day^-1^) | 1.19-58.8 | 0.84 |  |  |  |  |  | 1 | 0.17 | 0.18 | 0.22 | 0.28 |
| Temp.  (°C) | 11.8-27.4 | 0.14 |  |  |  |  |  |  | 1 | -0.52** | -0.44* | -0.26 |
| [NO3]  (µmol m^-3^) | 0-25.6 | 0.74 |  |  |  |  |  |  |  | 1 | 0.81*** | 0.82*** |
| [PO4]  (µmol m^-3^) | 0-0.7 | 0.75 |  |  |  |  |  |  |  |  | 1 | 0.75*** |
| [SiO3]  (µmol m^-3^) | 0.18-19.4 | 0.36 |  |  |  |  |  |  |  |  |  | 1 |


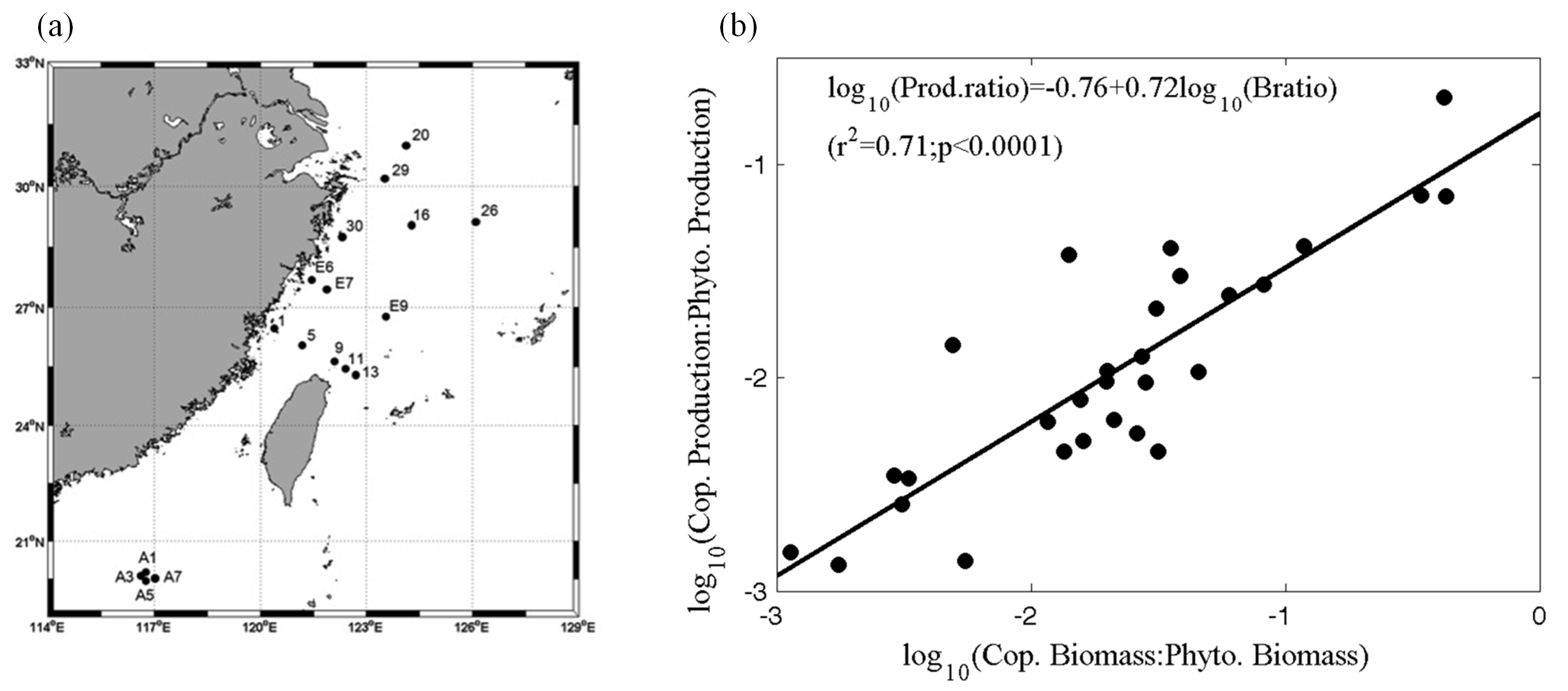


Figure C1. (a) Map illustraing sampling stations in the East China Sea and South China Sea. Some stations were sampled more than once. (b) Scatter plot illustrating the positive relationship between the copepods/phytoplankton production ratio versus the copepods/phytoplankton biomass ratio. The regression remains significant when data from the South China Sea are removed.


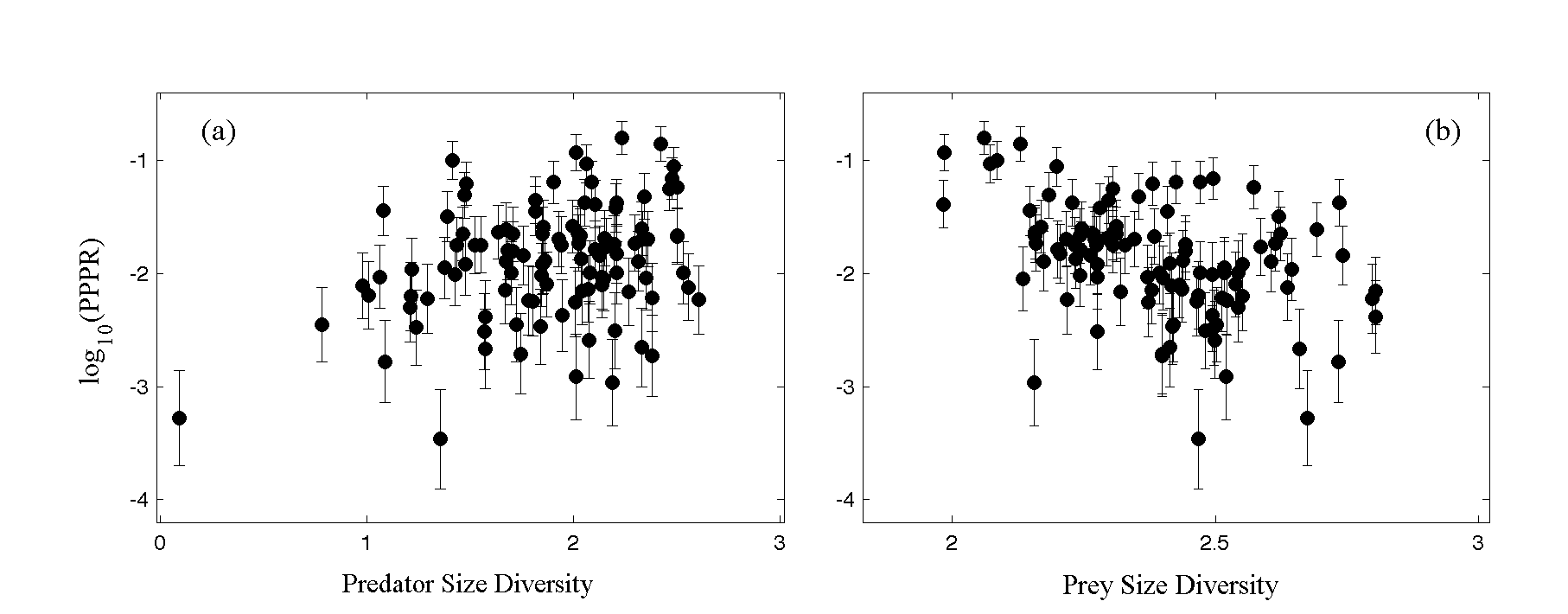


Figure C2. Relationships between log_10_(PPPR) versus (a) predator size diversity and (b) prey size diversity. Here, the log_10_(PPPR) was derived from the PPPR-PPBR relationship for only copepods (Fig. C1b). Error bars correspond to ± σ estimated from bootstrap.


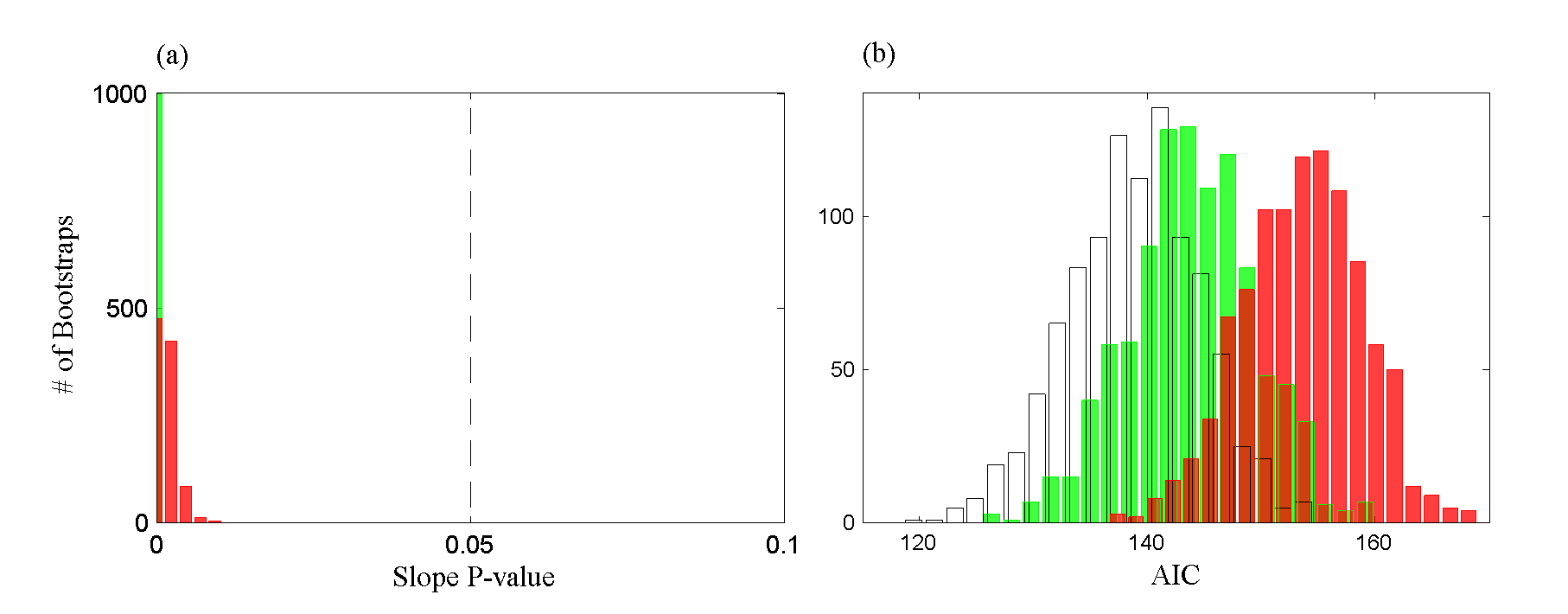


Figure C3. Results of the sensitivity analysis using PPBR as a proxy for PPPR considering error propagation. Panel (a) illustrates the histogram of p-values signaling significance of the slopes calculated for predator (red bars) and prey (green bars) size diversities as single explanatory variables of log_10_(PPPR). Here, the error propagation was estimated from the PPPR-PPBR relationship using the error calculated from the 1000 bootstraps. Likewise, panel (b) illustrates the histogram of Akaike information criterion (AIC) of the 1000 bootstraps of both models, and of the interaction of predator and prey size diversities (white bars).

References:

1. Huntley, M. E., Lopez, M. D. 1992 Temperature-dependent production of marine copepods: a global synthesis. *Am. Nat.* **140**, 201-242.

2. Gaedke, U., Straile, D. 1994 Seasonal-changes of trophic transfer efficiencies in a plankton food-web derived from biomass size distributions and network analysis. *Ecol. Model.* **75**, 435-445.

3. Yvon-Durocher, G., Montoya, J.M., Trimmer, M., Woodward, G.U.Y. 2011 Warming alters the size spectrum and shifts the distribution of biomass in freshwater ecosystems. *Global Change Biol.* **17**, 1681-1694. (doi:10.1111/j.1365-2486.2010.02321.x).

4. Ye, L., Chang, C.-Y., García-Comas, C., Gong, G.-C., Hsieh, C.-h. 2013 Increasing zooplankton size diversity enhances the strength of top-down control on phytoplankton through diet niche partitioning. *J Anim Ecol* **82**, 1052-1061. (doi:10.1111/1365-2656.12067).

5. Lin, K.Y., Sastri, A.R., Gong, G.C., Hsieh, C.H. 2013. Copepod community growth rates in relation to body size, temperature, and food availability in the East China Sea: a test of metabolic theory of ecology. *Biogeosciences* **10**, 1877-1892

6. Gong, G.-C., Shiah, F.-K., Liu, K.-K., Wen, Y.-H., Liang, M.-H. 2000 Spatial and temporal variation of chlorophyll a, primary productivity and chemical hydrography in the southern East China Sea. *Cont. Shelf Res.* **20**, 411-436.

7. Davison, A.-C., Hinkley, D. V. 1997 Bootstrap methods and their application. Series: Cambrige Series in Statistical and Probabilistic Mathematics (No. 1). 594 pp. Cambridge University Press. *ISBN-10: 0521574714.*

8. Brucet, S., Boix, D., Gascón, S., Sala, J., Quintana, X. D., Badosa, A., Søndergaard, M., Lauridsen, T. L., Jeppesen, E. 2009 Species richness of crustacean zooplankton and trophic structure of brackish lagoons in contrasting climate zones: north temperate Denmark and Mediterranean Catalonia (Spain). *Ecography* **32**, 692-702.

**Appendix D: Spatial autocorrelation**

Spatial autocorrelation of the response variables (i.e., Log_10_(PPBR), predator size diversity and prey size diversity) was estimated with the traditional Moran’s I measure. Tests were conducted by cruise and on the whole dataset, as our sampling strategy is spatio-temporally irregular. Only predator size diversity showed significant autocorrelation when considering all samples together (table D1). Thus, spatial autocorrelation is not a concern for Log_10_(PPBR) and prey size diversity.

Table D1. Results of analyses based on Moran’s I for each cruise and the whole dataset.

|  | Moran’s I (p-value) | | |
| --- | --- | --- | --- |
| Cruises (n) | Log_10_(PPBR) | Pred.S.D. | Prey S.D. |
| All (106) | 0.049 (0.50) | 0.25 (**0.02**) | 0.037 (0.59) |
| May 2009 (12) | -0.072 (0.82) | -0.034 (0.47) | 0012 (0.14) |
| July 2009 (22) | -0.100 (0.54) | 0.073 (0.17) | -0.009 (0.68) |
| April 2010 (9) | 0.026 (0.08) | -0.159 (0.49) | -0.156 (0.54) |
| May 2010 (5) | -0.169 (0.84) | -0.015 (0.14) | -0.294 (0.53) |
| July 2010 (17) | -0.0213 (0.57) | 0.019 (0.23) | -0.035 (0.71) |
| Sept. 2010 (3) | -0.415 (0.75) | -0.169 (0.53) | -0.087 (0.33) |
| July 2011 (4) | -0.173 (0.39) | -0.293 (0.72) | -0.195 (0.63) |
| Aug. 2011 (7) | -0.018 (0.29) | 0.003 (0.20) | -0.009 (0.24) |
| Oct. 2012 (9) | -0.179 (0.44) | -0.009 (0.27) | -0.139 (0.77) |
| July 2012 (12) | -0.043 (0.79) | 0.015 (0.52) | -0.154 (0.58) |
| May2013 (6) | -0.217 (0.58) | -0.209 (0.57) | -0.097 (0.42) |

For predator size diversity, distanced-based Moran’s eigenvector mapping (db-MEM or PCNM) [1] was applied in order to individualize spatial structures significantly shaping the predator size diversity. Distance-based MEM consists of dissecting the spatial structure into orthogonal spatial variables based only on distance among close neighbours. Out of 15 orthogonal vectors (i.e., independent) representing positive spatial autocorrelation, 4 vectors were individualized without prior linear detrending (adj. R^2^=0.33). In order to test if spatial autocorrelation affects our result, we added to the selected most parsimonious model the 4 spatial vectors representing the spatial autocorrelation of the predator size diversity. The relationship was still significant, and thus spatial autocorrelation did not affect the conclusions of this study, albeit caution imposes related to sampling strategy (Table D2).

Table D2. Results of the most parsimonious LMM explaining Predator size diversity accounting for spatial autocorrelation (4 independent vectors).

| **Response variable: Predator Size Diversity** | | | | | |  |
| --- | --- | --- | --- | --- | --- | --- |
| Model:  AICc=90.75 | Slope | | | |  | |
|  | Estimate | SE | *t* value | p-value | d.f. | |
| SiO_3_ | -0.19 | 0.064 | -2.92 | 0.004** | 61 | |
| Space1 | 0.91 | 0.377 | 2.42 | 0.018* | 61 | |
| Space2 | 1.15 | 0.441 | 2.62 | 0.011* | 61 | |
| Space3 | -0.85 | 0.362 | -2.36 | 0.021* | 61 | |
| Space4 | -1.23 | 0.354 | -3.48 | 0.0009*** | 61 | |

References:

1. Borcard, D., Legendre, P., Avois-Jacquet, C., Tuomisto, H. 2004 Disecting the spatial structure of ecological data at multiple scales. *Ecology* **85**, 1826-1832.

**Appendix E: Basic statistics and pair-wise relationships**

Table E1. Basic statistics for the variables used in this study. Nutrient concentrations are depth-weighted values integrated above the mixed layer depth (MLD). Note, that for all correlation analyses, nutrient concentrations are log-transformed.

| VARIABLES | Mean±SD | Min:Max | CV |
| --- | --- | --- | --- |
| Log_10_(PPBR) | -1.57±0.70 | -3.75:-0.05 | 0.45 |
| Predator size diversity | 1.88±0.45 | 0.09-2.61 | 0.24 |
| Prey size diversity | 2.39±0.18 | 1.98-2.80 | 0.08 |
| SST (°C) | 24.39±3.71 | 11.79-29.73 | 0.15 |
| NO_3_ (µmol m^-3^) | 3.02±5.36 | 0-25.36 | 1.77 |
| SiO_3_(µmol m^-3^) | 6.16±5.96 | 0.42-27.05 | 1.27 |
| PO_4_ (µmol m^-3^) | 0.21±0.26 | 0-1.28 | 0.97 |


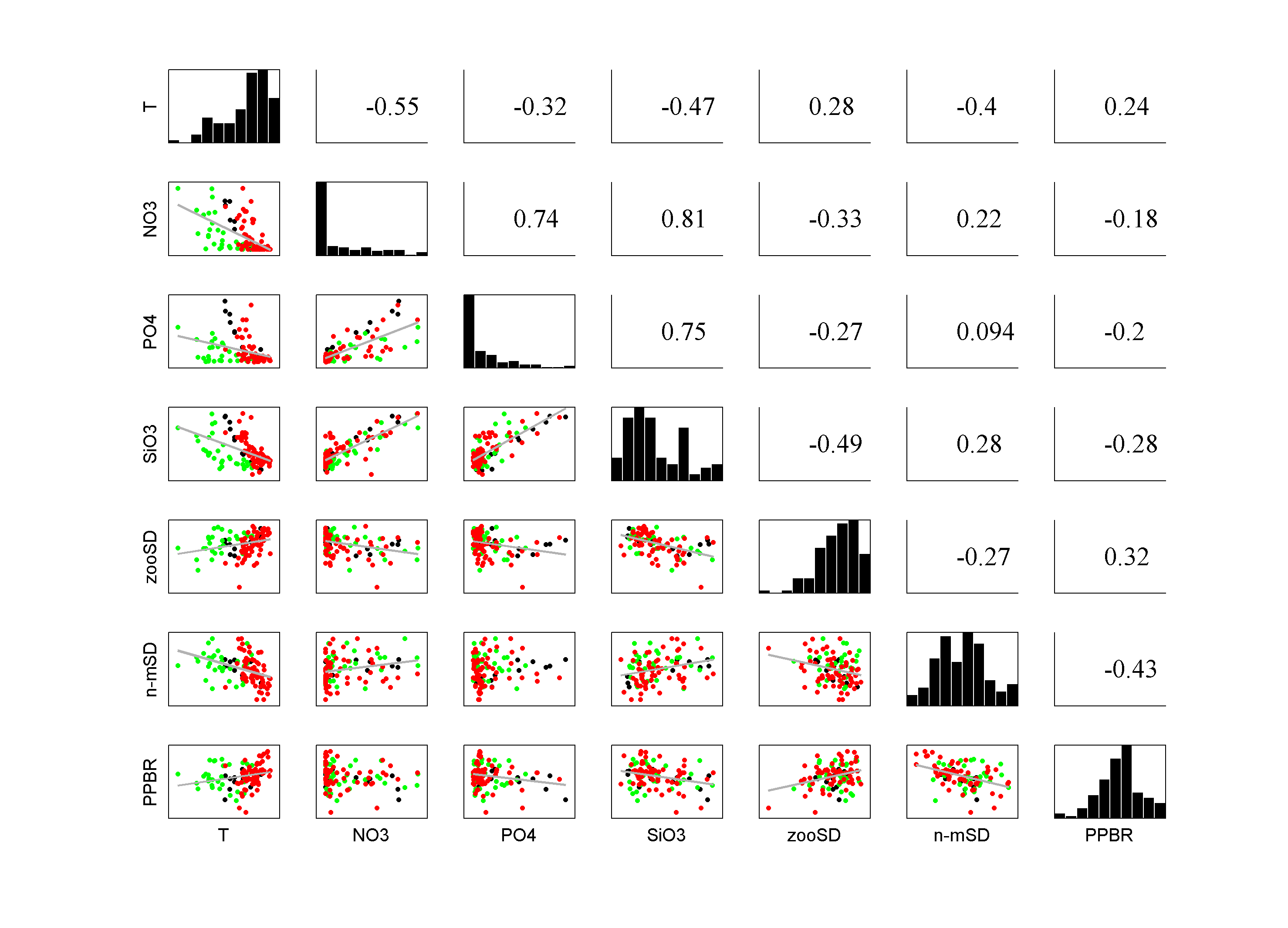


Fig. E1. Pair-wise relationships. Regression lines are only depicted for significant relationships. Colors represent the sampling season (black: autumn, red: summer, green: spring). Histograms represent the distribution of each variable, and numbers correspond to pair-wise Pearson correlation.

**Appendix F: Model rankings according to their AICc**

Model rankings were performed with the dredge function of R package MuMln. Models are ranked from the best to the worst fitting according to their Akaike weight ($\omega$). The Akaike weight of each model ($\omega_{i}$) corresponds to:

$$\omega_{i}=\frac{\exp\left[ -\frac{1}{2}\Delta_{i} \right]}{\sum_{i=1}^{m} \exp\left[ -\frac{1}{2}\Delta_{i} \right]}$$

where $\Delta_{i}$ corresponds to:

$\Delta_{i}=\mathrm{AIC}_{i}-\mathrm{AIC}_{\min}$

The greater the Akaike weight of a model, the higher the probability of being the best model.


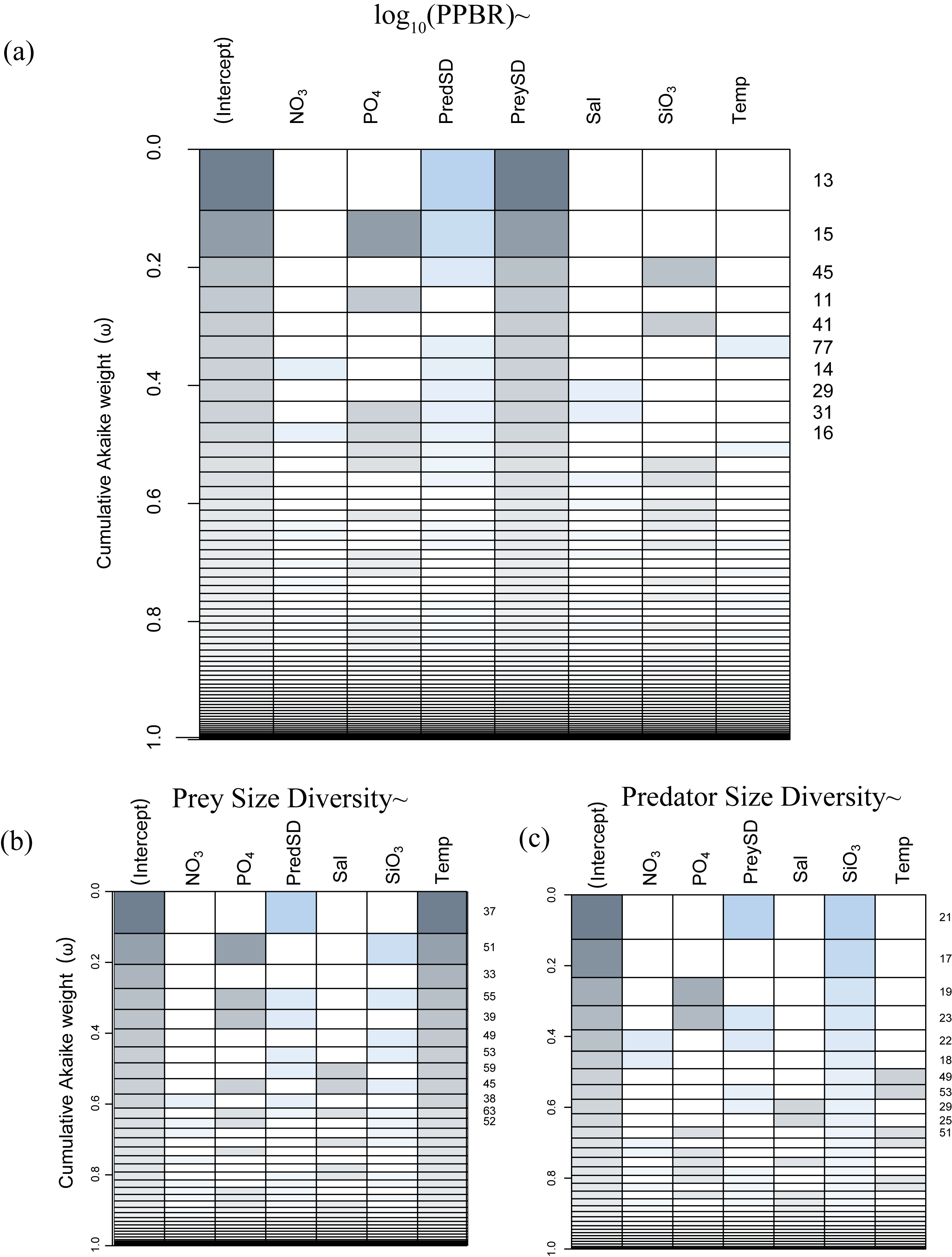


Fig F1. Cumulative Akaike weights of ranked models explaining (a) biomass transfer efficiency, (b) prey size diversity, and (c) predator size diversity. Interaction of explanatory variables in the most parsimonious model was also tested accordingly. Color indicates the variables (columns) constituting each ranked model (rows).


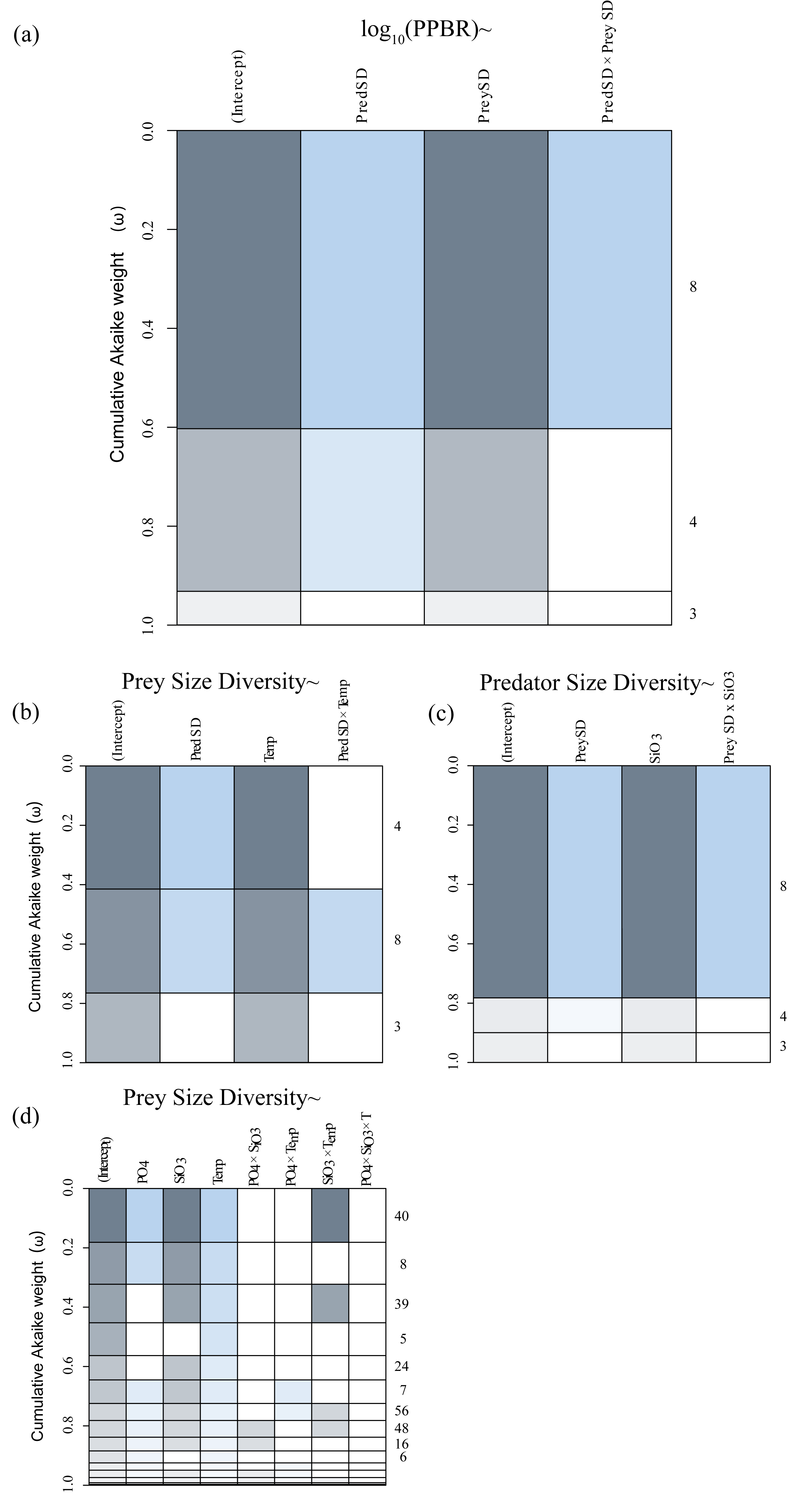


Fig F2. Cumulative Akaike weights of ranked interactions in the most parsimonious models explaining (a) biomass transfer efficiency, (b) prey size diversity, and (c) predator size diversity with predator and prey size diversities included, and (d) prey size diversity without predator size diversity as one of the explanatory variables. Color indicates the variables (columns) constituting each ranked model (rows).

**Appendix G: Mean size ratio and predator-prey mean size as explanatory variables for trophic transfer efficiency**

We hypothesized that size-based diet niche partitioning is the main mechanism linking predator size diversity to trophic transfer efficiency. In order to further test this hypothesis, we considered how much of the variation in trophic transfer was explained by the predator-prey size diversity ratio relative to predator-prey individual mass ratio (PPMR) and the mean size of predators. The PPMR and mean size reflect how the increasing presence of large predators enhances trophic transfer due to their greater effectiveness at capturing and manipulating prey.

The median body size (individual biovolume; mm^3^), of both predators and prey, was estimated for all of the 106 samples instead of the mean to account for skewness in the size distribution. We defined predator-prey size ratio (PpSR) as the ratio of median body sizes of predators and prey in each sample, and used this index as a proxy for the more commonly used community PPMR index (because we lacked size-based trophic-interaction data). We then investigated if PpSR or the median size of predators performed better than predator size diversity at explaining the trophic transfer efficiency (predator/prey biomass ratio as a proxy; log_10_(PPBR)). Our results indicate that both PpSR and the median size of predators (and prey) represented a poorer fit than size diversities (i.e., higher AICc) (Table F1).

In addition, we also compared models including predator size diversity, predator median size and their interaction. The most parsimonious model corresponded to the sum of predator size diversity and median size explaining log_10_(PPBR) (Fig. F1). This result stands in contrast to the hypothesis that trophic transfer efficiency varies entirely with the mean body size of predators *per se*.

Table G1. Results of LMMs explaining variation in transfer efficiency (log_10_(PPBR)) when the predator to prey size diversities ratio, the predator-prey median size ratio (PpSR) and predator and prey median sizes were used as explanatory variables. The values in brackets represent the difference between AICc values for comparing size diversity model with its corresponding analog (e.g. comparison of the models with predator size diversity versus predator mean size).

| **Response variable: log_10_(PPBR)** | | | | | |  |
| --- | --- | --- | --- | --- | --- | --- |
| 1 Explanatory Variable | AICc | Slope | | | |  |
|  |  | Estimate | SE | *t* value | p-value | d.f. |
| Pred. size div./Prey size div. | 214 | 1.345 | 0.302 | 4.457 | <0.0001*** | 65 |
| PpSR | 225 (+11) | 1.4 10^-8^ | 5 10^-9^ | 2.729 | 0.008** | 65 |
| Predator median size | 228 (+7) | 0.669 | 0.329 | 2.033 | 0.046^.^ | 65 |
| Prey median size | 219 (+9) | -4.4 10^7^ | 1.2 10^7^ | -3.812 | 0.0001*** | 65 |

**
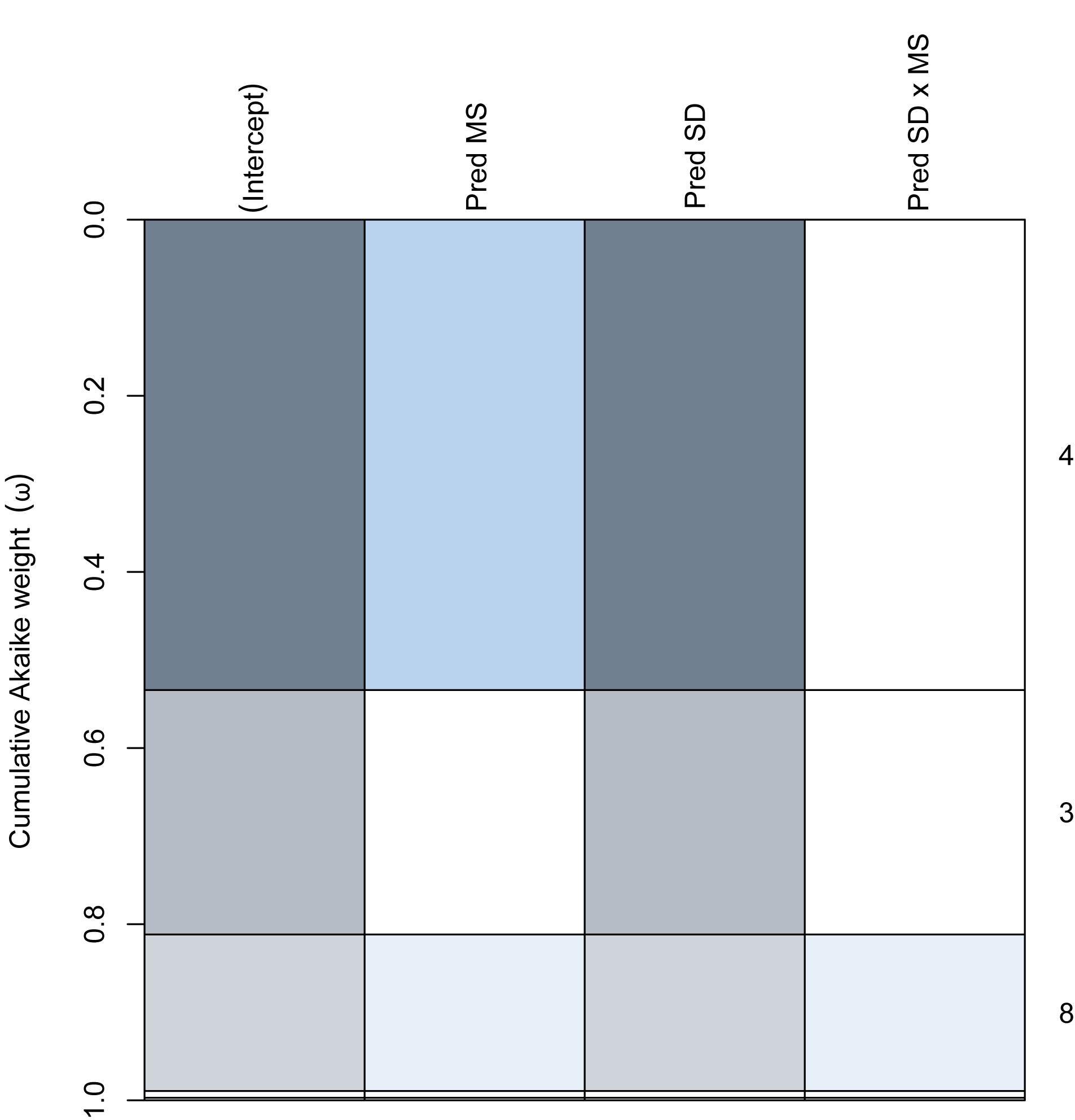
**

Fig G1. Cumulative Akaike weights of ranked models explaining log_10_(PPBR) with predator size diversity and median size as well as their interaction tested as explanatory variables. Color indicates the variables (columns) constituting each ranked model (rows).


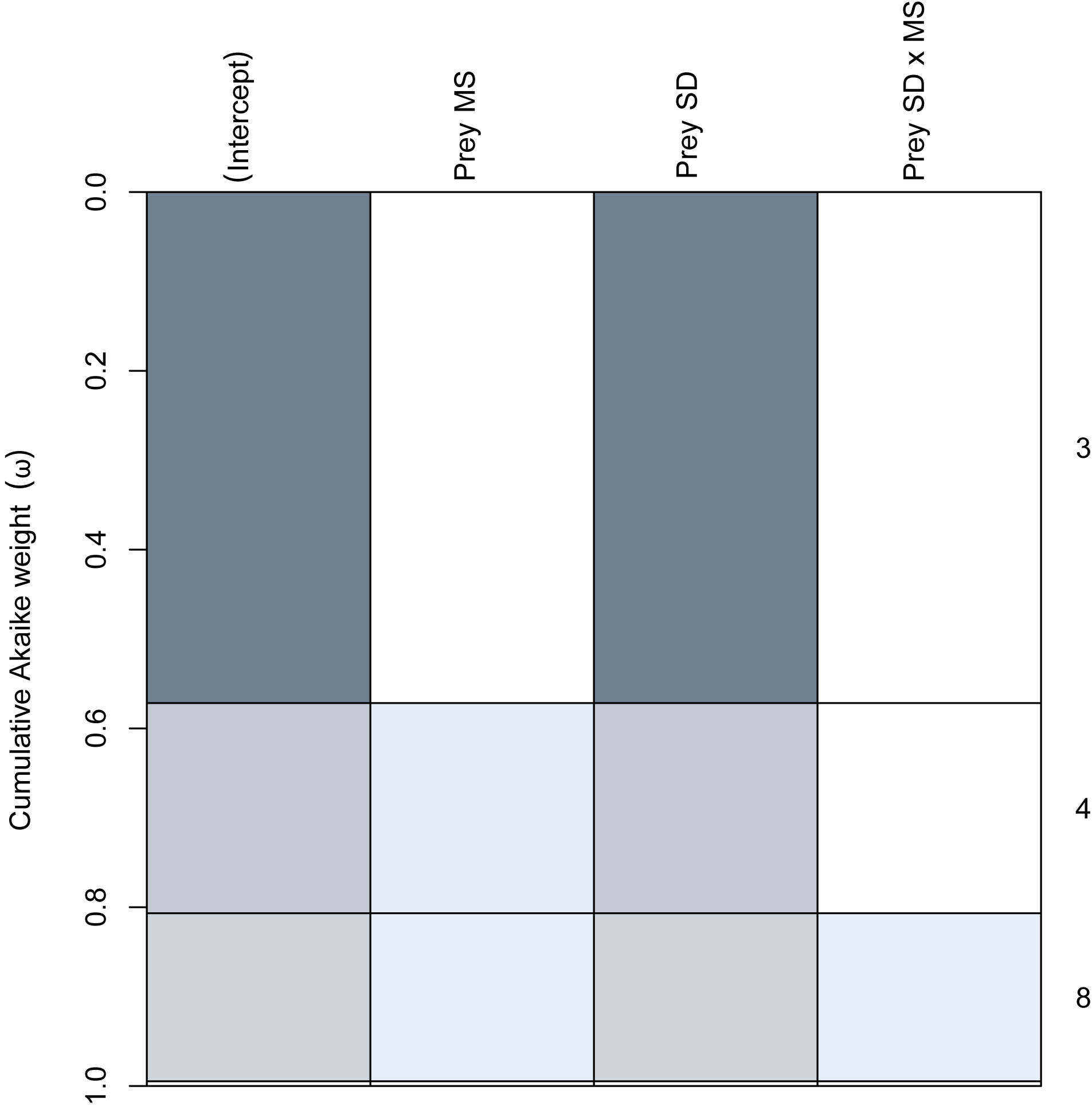


Fig G2. Cumulative Akaike weights of ranked models explaining log_10_(PPBR) with prey size diversity and median size as well as their interaction tested as explanatory variables. Color indicates the variables (columns) constituting each ranked model (rows).
